# Fungal Chitosan-Derived Biomaterials Modified with *Kalanchoe pinnata* as Potential Hemostatic Agents—Development and Characterization

**DOI:** 10.3390/polym13081300

**Published:** 2021-04-15

**Authors:** Julia Radwan-Pragłowska, Łukasz Janus, Marek Piątkowski, Aleksandra Sierakowska, Tomasz Galek, Ernest Szajna, Dariusz Bogdał, Mirosław Tupaj

**Affiliations:** 1 Department of Biotechnology and Physical Chemistry, Faculty of Chemical Engineering and Technology, Cracow University of Technology, Warszawska 24 Street, 31-155 Cracow, Poland; lukasz.janus@doktorant.pk.edu.pl (Ł.J.); marek.piatkowski@pk.edu.pl (M.P.); a.sierakowska3530@doktorant.pk.edu.pl (A.S.); pcbogdal@cyf-kr.edu.pl (D.B.); 2 Faculty of Mechanics and Technology, Rzeszow University of Technology, Kwiatkowskiego 4 Street, 37-450 Stalowa Wola, Poland; t.galek@prz.edu.pl (T.G.); mirek@prz.edu.pl (M.T.); 3 Nano Prime, Metalowców 25, 39-200 Dębica, Poland; e.szajna@uce.com.pl

**Keywords:** chitosan, hydrogel, biomaterials

## Abstract

Massive blood loss is still a great challenge for modern medicine. To stop the hemorrhage during the surgery or after injury apart from suturing or electrocoagulation, the most efficient method of hemostasis restoration is the use of hemostatic agents. Although there are numerous products on the market, there is still a need for biomaterials that are capable of fast and efficient bleeding management without affecting wound closure or embolism. Chitosan is known for its hemostatic activity; however, its quite poor mechanical properties and heterogenous chemical composition still needs some improvements to become superior compared to biological adhesives. The following study deals with the preparation and evaluation of chitosan-derived natural biomaterials containing *Kalanchoe pinnata* extract with the potential application as a blood-clotting agent. The materials were obtained under microwave-assisted conditions in two different forms (granules/dressing), whose chemical structure and morphology were studied. Their antioxidant properties have been proven. The chitosan-derived hemostatic agents exhibited superior blood sorption abilities and lack of cytotoxicity to L929 mouse fibroblasts. The study also showed the differences in biological properties depending on their preparation method. The potential mechanism of action was proposed as well as their potential in hemostasis revival.

## 1. Introduction

One of the biggest challenges for the modern medicine is massive blood loss, which is responsible for the highest number of deaths resulting from various injuries. Until now, no functional, Food and Drug Administration-approved artificial blood substitutes have been developed. Thus, the ability of fast and efficient inhibition of bleeding is an issue of a great importance. Currently, the most popular methods of hemostasis restoration include the use of polymeric sutures, electrocoagulation procedures, or application of hemostatic agents (HemA). The choice of HemA is especially understandable in the case of hemorrhages in the area of sensitive body parts such as nerves or medullary bone. Such materials are also willingly used in patients suffering from various hemostatic abnormalities. HemA minimizes the risk of peri-operative bleeding, decreases surgery duration, and provides support for the veins and arteries [1,2,3]. Importantly, hemostatic agents can be used not only by medics but also by individuals or military. HemA are commonly used in army in the battlefield when the bleedings come as a result of shooting or explosion. In such cases, HemA in the form of a powder or a plug is placed inside the deep wound and helps to stop the blood outflow from the veins before the patient’s transport to the medical unit.

HemA can be divided into two main groups. The first one includes inorganic and non-resorbable materials, which are mostly zeolites. Due to their porous structure, they are capable of blood sorption; however, their use is associated with the risk of heat generation, leading to inflammatory reactions or even thermal injuries of the surrounding tissues, which is their main flaw. In addition, they must be removed from the wound site. Other examples include HemA based on bone wax. The second group comprises biodegradable materials, which are mostly prepared from various natural polymers such as microfibrillar collagen, gelatin, oxidized regenerated cellulose, polysaccharidic microspheres, and others [1,4,5,6,7,8]. These are mainly applied in the form of a dry matrix with a porous structure. Their mode of action is based on their capability of blood plasma sorption leading to the hyperconcentration of coagulation proteins as well as platelets. They undergo bioresorption process up to 10 weeks after application. However, their physicochemical and biological properties may affect the wound closure process, especially due to the significant volume increase as a result of swelling with blood. Alternatively, biological hemostatic agents such as thrombin, fibrin/fibrinogen, or tranexamic acid are used. Bovine-origin thrombin acts as a natural catalyst for hemostasis restoration, since it helps to convert fibrinogen into fibrin. In turn, fibrin sealants can form a fibrin clot and induce hemostasis in only few minutes as a result of physiological cascade imitation. These biomaterials are used especially during cardiovascular surgeries associated with a high risk. Another group of HemA consists of adhesives that stop the bleeding only due to tissues sealing. Importantly, they do not absorb blood or affect coagulation cascade. Such biomaterials are based on polymers including poly(ethylene glycol), which turns into hydrogel after contact with blood or animal-derived albumin crosslinked with glutaraldehyde. Due to the fast mode of action (tissue sealing after 5–30 s), they are applied during surgeries dealing e.g., with aortic dissections. Sometimes, so-called dual HemA also can be found on the market. They provide support for natural clot formation and induce blood coagulation.

When choosing appropriate HemA, one must take under consideration the mode of action (biologically passive or active), form (powder, glue, patch), and possible side effects including allergic reactions coming from animal-derived substrates, embolism, hypotension, bloodborne disease, infection, immune-mediated coagulopathy, vascular thrombosis, anaphylaxis, and others. Since all of the aforementioned biomaterials used for hemostasis restoration have some downfalls or limitations in the scope of their application, there is still a great need for novel hemostatic agents’ development characterized with improved both physicochemical and biological properties [1,9].

One of the most promising raw materials for HemA preparation is chitosan (CS), which is a natural polymer obtained from chitin—the second most abundant polysaccharide after cellulose. This polycation can be produced via the deacetylation of chitin using various biowaste from the food industry, agriculture, and others [9,10,11,12]. Commercially, CS is mostly prepared as a result of treating chitin with NaOH solution, leading to the removal of *N*-acetyl groups from its units. This process carried out on an industrial scale is proceeded under harsh conditions and relies on the use of shrimps, crabs, lobsters or squids’ exoskeletons as a substrate, which are washed, dried, and homogenized. The first step of CS commercial production involves chitin demineralization performed using hydrochloric acid followed by deproteinization with sodium hydroxide of low concentration. To remove the color, a bleaching process is carried out with hydrogen peroxide. Finally, pre-treated chitin undergoes the deacetylation via treating with 40–50% NaOH solution. The process is finished when the polymer contains at least 60% of glucosamine units in its structure. Alternatively, CS can be prepared via the biological route using bacteria-produced organic acids and specific enzymes such as proteases or chitin deacetylases. The enzymatic method is considered as environmentally friendly and enables preparation of the product with more uniform physicochemical properties such as average molecular mass, viscosity, and deacetylation degree. CS exhibits a significant resemblance to the naturally occurring extracellular matrix (ECM) components. This can be assigned to its chemical structure. Moreover, it is proven to be non-toxic and is well known for its biocompatibility. It undergoes biodegradation and exhibits antioxidant properties. It contains free hydroxyl and amino groups; thus, it may undergo various modifications, especially crosslinking. Moreover, it can be used for various enzymes immobilization such as laccase due to the possibility of amide bonds formation with carboxyl groups of the biomolecules. CS is also frequently used for the preparation of smart materials for various applications such as catalysis, remediation, agriculture, food packaging, and others. When designing a new, CS-based composite, polymer content must be chosen very carefully, since it affects many parameters of the final product such as active substance loading capacity [12]. It dissolves in aqueous solutions with pH below 6.3. Amino groups present in glucosamine (deacetylated units) are responsible for many attractive properties of CS such as antibacterial activity or chelating capability. CS may also interact with cell membrane components and has a hemostatic effect. It enhances cells adhesion and proliferation. CS may form physical and chemical hydrogels capable of aqueous solutions sorption. Native CS can be used as a powder, coated bandage, or patch to prevent bleeding. To increase its mechanical properties and enhance ability to hemostasis restoration, the polymer can be crosslinked to form three-dimensional, spacious structure. However, this leads to the loss of free amino groups, which may have a negative effect on its overall biological performance [9,13,14,15,16,17].

CS hemostatic agents’ mode of action in the first way is based on its ability to absorb blood plasma and interact with erythrocytes, leading to their coagulation. The polymer swells with aqueous fraction of the blood, therefore concentrating its components, which take place in hemostasis reviewing. CS may enhance red blood cells adhesion and coagulation due to the absorption of proteins present in blood such as fibrinogen and helps in clot formation, yet still it does not explain its hemostatic activity [9]. This polysaccharide induces platelets adhesion followed by aggregation and finally activation, which is crucial during hemorrhage managing. It has been shown that CS may affect platelets spreading and adhesion stability, since it can generate various intracellular signal reactions. For example, it can activate glycoprotein IIb/IIIa or discharge thromboxane A2/ADP. In addition, some studies prove that platelets after direct contact with CS exhibit a higher expression of integrin α2β3. Finally, due to its ability of ions’ chelation, CS may bind Ca^2+^ ions that are responsible for actin skeleton activation [9,10]. From this point of view, it must be acknowledged that to provide sufficient hemostatic activity of CS-based HemA, two main factors must be taken under consideration, namely sorption abilities, which are a result of the hydrophilic nature of the material due to certain functional groups such as hydroxyl, amino, or carboxylic as well as porous, open-cell, spatial architecture, which provides the possibility of water molecules migration inside the polymeric network, thus increasing swelling capability [4,5,6,7,8,9].

To enhance the biological activity of the biomaterials, various methods can be applied. These include blends formation with other polymers and biopolymers or inorganic substrates as well as doping with nanoparticles. Nevertheless, the addition of an extra component may deteriorate the biodegradation/bioresorption rate of the material or affect its mechanical durability. CS by itself exhibits some interesting features. One of them is antioxidant activity and positive impact on cells proliferation. Bioactivity is a highly desired property for modern HemA. *Kalanchoe pinnata* is a Madagascar-origin plant whose leaves and juice show strong anti-inflammatory, bactericidal, fungicidal, and virucidal activity. It perfectly regenerates all kinds of skin lesions. Moreover, it is known for its antioxidant and hepatoprotective properties and thus constitutes a great natural alternative to currently applied supporting drugs [18,19,20,21,22,23,24,25,26].

In this paper, a novel strategy for bioactive HemA preparation is presented using *Aspergillus niger*-derived CS as a main substrate. The biopolymer was chemically crosslinked with amino acids followed by modification with *Kalanchoe pinnata* leaves extracts. Ready biomaterials were investigated over their chemical structure with the use of the Fourier transform infrared spectroscopy (FTIR) method, morphology (e.g., using Scanning Electron Microscopy), mechanical durability, swelling properties, and antioxidant activity. Finally, the products were confirmed to be non-toxic to the L929 mouse fibroblasts cell line.

## 2. Materials and Methods

### 2.1. Materials

For the experiments, fungal chitosan from Aspergillus niger was used with the average molecular mass 890,000, deacetylation degree ≥ 90%, ash content ≤ 1.0%, viscosity 100–300 cps, water content ≤ 8.0%, As ≤ 1 mg/kg, Pb ≤ 0.5 mg/kg, Hg ≤ 0.1 mg/kg, particle size ≤ 100 mesh, E. coli microbial counts: absent, *Salmonella* spp.: absent, yeasts and molds: ≤100 CFU/g purified from glucan purchased from PolAura, Dywity, Poland. L-aspartic acid, L-glutamic acid, glycerin, and 1,2-propanediol were purchased from PolAura, Dywity, Poland. *Kalanchoe pinnata* extract was purchased from Ekamedica, Kozy, Poland. Simulated body fluid (SBF), Dulbecco’s Modified Eagle’s Medium, trypsin, fetal bovine serum, phosphate buffer solution, and penicillin/streptomycin solution (10%) were purchased from PolGen. Multiwhole plates, T-75 flasks, sterile pipette tips, probes, and falcons were purchased from GenoPlast, Rokocin, Poland. XTT (2,3-Bis-(2-Methoxy-4-Nitro-5-Sulfophenyl)-2H-Tetrazolium-5-Carboxanilide) assay (Roche), L929 mouse fibroblasts cell line, and lyophilized blood for scientific use only were purchased from Sigma-Aldrich, Poznań, Poland. Rhodamin, ethanol, mehtanol, and gallic acid were purchased from POCH, Gliwice, Poland. Folin–Ciocalteu reagent was synthesized by standard procedure [27,28].

### 2.2. Methods

#### 2.2.1. Samples’ Preparation

**Figure 1:**
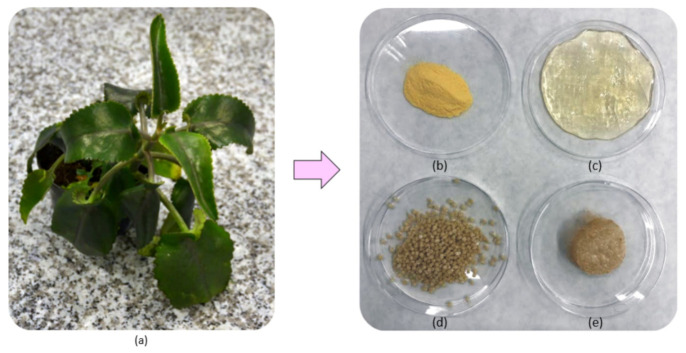
Photographs of (**a**) *Kalanchoe* *pinnata* plant used for extracts preparation (**b**) native chitosan (CS) (**c**) crosslinked hydrogel; (**d**) ready hemostatic agent in the form of beads; (**e**) hemostatic agent in the form of a patch.

To obtain chemically crosslinked chitosan-based hydrogels, fungal CS has been dissolved in 5% solution of acetic acid, precipitated with 5% sodium hydroxide solution, and lyophilized. To prepare the reaction mixture, 0.5 g of the precipitated CS was added in portions to the aqueous solution of L-aspartic and L-glutamic acid (pH < 6.3). Then, 30 mL of 1,2-propanediol were added to enable microwave radiation absorption. The glycol acted as a high boiling solvent. The reaction vessel (200 mL volume) was placed in the microwave reactor for 3 min with power set on 800 W. After water evaporation, the crosslinking process has occurred, resulting in the CS color change. Then, the product was separated from the 1,2-propanediol and washed out from unreacted amino acids residues using distilled water until pH above 6 was achieved. To immobilize *Kalanchoe pinnata* extracts, each of the natural solutions was added to the swelling medium (distilled water). The samples (hydrogels) were immersed into the swelling solutions. The products were prepared in two different forms, namely a patch and beads. To prepare potential HemA in the form of a patch, the swollen hydrogels were placed in polypropylene boxes and lyophilized. To obtain hemostatic agents is the form of beads, the precipitated CS was dissolved in the 5% solution of amino acids and added dropwise to the NaOH solution using a syringe pump. Before lyophilization, cryoprotectant (glycol) was added to preserve the spherical structure. Samples description is given in the Table 1, while the components are presented in the Figure 1.

For the experiments, 4 different *Kalanchoe pinnata* extracts were used. Commercial extract was added to the samples without any pre-treatment in the amout of 1% *w/w*. To prepare fresh extracts, fresh leaves were cut, frozen, and lyophilized using a freeze dryer (Alpha 1–4, Donserv, Warszawa, Poland). Then, 1 g of lyophilized leaves was placed in 50 mL of ethanol solutions (50% and 95%). The extraction was carried out for 24 h under dark conditions to prevent photodegradation. Additionally, the plant extract prepared in 95% ethanol was subjected to ultrasounds using an ultrasonic bath (1 h).

#### 2.2.2. Fourier Transform Infrared Spectroscopy (FTIR) Study

FTIR analysis was carried out using a Thermo Nicolet Nexus 470 FT-IR spectrometer from Thermo Fisher Scientific, Waltham, MA, USA equipped with an attenuated total reflection (ATR) diamond adapter. For the analysis, 50 mg of each dried sample was used. The FTIR technique is used for determining the deacetylation degree. This method uses a characteristic absorption band corresponding to the amide group of the *N*-acetylglucosamine unit and a reference band that occurs in both glucosamine and *N*-acetylglucosamine units. These bands are visible at wavenumbers of 1655 cm^−1^ and 2870 cm^−1^. They correspond to the amounts of amide and hydroxyl groups in chitosan, respectively. The degree of deacetylation is calculated according to the equation:

(1) $$\mathrm{DD}\%=100-\frac{A_{1655}}{A_{2870}}\times \frac{100}{1.33}$$

where:

DD—deacetylation degree, %.
A_1655_—band absorption field at the wavenumber equal to 1635 cm^−1^.
A_2870_—band absorption field at the wavenumber equal to 3324 cm^−1^.

#### 2.2.3. Viscosity Study

The viscosity of the samples and raw CS was determined using Brookfield RV rotational viscometer DV-II+ Pro. Viscosity of the native CS was measured by preparing 1% polymer solution in acetic acid. Samples after crosslinking (fresh hydrogels) and samples after lyophilization (ready products) were swollen with distilled water. During the experiments, various spindles were used that enabled the evaluation of crosslinked samples.

#### 2.2.4. Morphology Study

The samples were investigated by the Scanning Electron Microscopy (SEM) method. SEM images were prepared using a high-resolution MIRA Scanning Electron Miscroscope purchased from Tescan, Brno, Czech Republic. All samples were in a dried form. Samples’ roughness was determined using Fiji image J software. The morphology of swollen samples was investigated using an inverted microscope (Delta Optical, Zielona Góra, Poland) and an epifluorescence adapter. For the analysis, materials were swollen with aqueous solution of rhodamine.

#### 2.2.5. Natural Extracts’ Properties Study

Plant extracts were verified over their optical properties by preparing methanol solutions (100× dilution), which were placed in quartz cuvettes (1 cm optical length), and UV-Vis spectra were collected in the range between 200 and 1000 nm using a UV-Vis Agilent 8453 diode-array spectrophotometer. Fluorescence spectra were collected in the range between 620 and 780 nm using a Jasco FP-750 spectrofluorimeter. The excitation wavelength was 365 nm, whereas the optical slit for excitation and emission was 5 nm. To determine phenolic and polyphenolic compounds content, the colorimetric method was used. For this purpose, Folin–Ciocalteu reagent was used. To proceed with the test, gallic acid as a standard was applied. The content of polyphenolic compounds was presented as µg per 1 mL of an extract. The test principle is based on the color change to dark blue. The measurements were performed at 750 nm. As a medium, ammonium buffer solution (pH = 10) was used [27,28].

Antioxidant properties study were carried out by the DPPH (2,2-diphenyl-1-picrylhydrazyl) method. Test duration was 1 h. The spectra were collected at 517 nm using UV-Vis spectrophotometer. For the experiments, freshly prepared DPPH solution in 95% ethanol with the concentration of 25 mg/L was used, which was stored under low temperature (5 °C) in the darkness.

#### 2.2.6. Swelling Properties Study

Swelling properties studies were carried out using three different swelling media, namely distilled water, SBF, and blood liophylizate, which was subjected to a rehydratation process. During the tests, each lyophilized sample was weighed, placed in the medium for 5 min, and weighed again. The swelling degree (SD) was calculated using following Equation (1):

SD = Ws − Wd/Wd·100%(2)

where:

SD—swelling degree, %.
Wd—weight of the dried sample, g.
Ws—weight of the swollen sample, g.

#### 2.2.7. Mechanical Properties Study

Mechanical durability of the biomaterials was determined by tensile strength (TS) parameter measurement. All samples were prepared in the shape of a so-called “dog bone” according to PN-EN ISO 527:1998 standard for plastics mechanical properties study. For this purpose, swollen samples were freeze-dried in the molds printed using a 3D printer (Ender, Warsaw, Poland) using poly(lactic acid) filament. Dried and shaped samples were swollen with SBF and had a thickness of 4.0 mm, overall length of 150 mm, and measuring part width of 10 mm.

#### 2.2.8. Cytotoxicity Study

Cytotoxicity was evaluated by quantitative and qualitative methods according to ISO 10993 standard for biomedical devices. For this purpose, L929 mouse fibroblasts were cultured under standard conditions (high humidity, 5% CO_2_, 37 °C). The cell culture medium was changed every 24 h. Cells’ proliferation assay based on the tetrazolium salt reduction (XTT assay) was carried out according to the producer’s protocol (Roche) after 48 h. The colorimetric reaction was investigated using a microplate reader at 450 nm. To evaluate cytotoxcicity by direct method, the cells were seeded inside the 24-well plates containing 10 mg of each sample. Their morphology was observed under an inverted microscope equipped with an epifluorescence adapter (Delta Optical, Zielona Góra, Poland) after 72 h of culture.

All experiments were performed in at least triplicate. Statistical analysis was performed by Excel software, and a *p* < 0.05 value was found to be statistically significant.

## 3. Results

### 3.1. Natural Extracts Investigation

**Figure 2:**
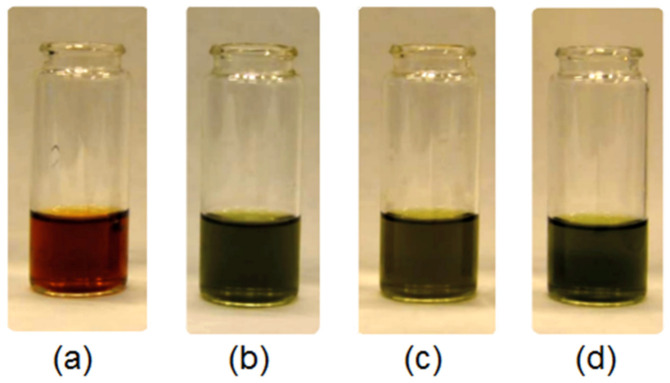
Photographs of the *Kalanchoe pinnata* extracts: (**a**) commercially available extract; (**b**) plant extract prepared using 50% ethanol solution; (**c**) plant extract prepared using 95% ethanol solution; (**d**) plant extract prepared using 95% ethanol solution and ultrasounds.

**Figure 3:**
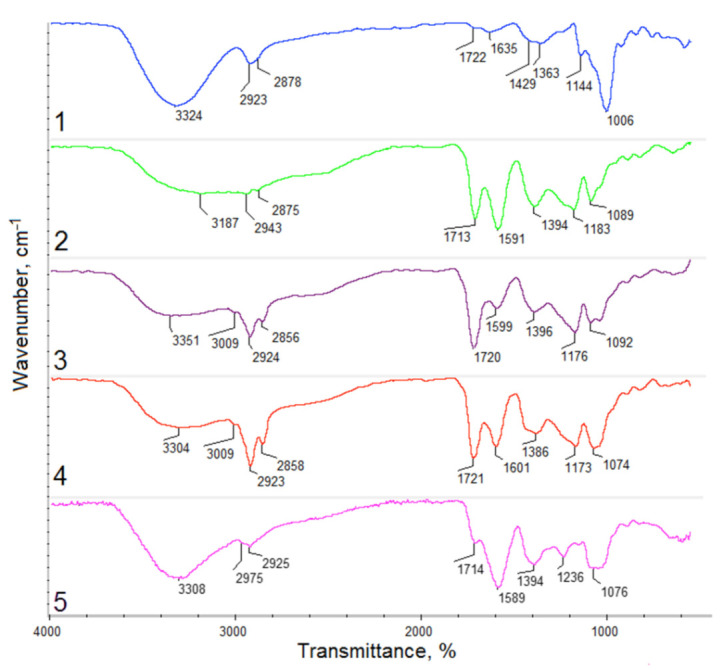
FTIR spectra of (**1**) *Kalanchoe pinnata* leaf; (**2**) commercially available extract; (**3**) plant extract prepared using 50% ethanol solution; (**4**) plant extract prepared using 95% ethanol solution; (**5**) plant extract prepared using 95% ethanol solution and ultrasounds.

Photographs of the *Kalanchoe pinnata* extracts can be found in Figure 2. Figure 3 shows FTIR spectra of the *Kalanchoe pinnata* leaf, which is commercially available, as well as self-made plant extracts. Results are given in Table 2. The spectrum of the lyophilized and ground leaves shows bands typical for polysaccharides at 3324 cm^−1^ coming from hydroxyl groups and 2923 cm^−1^ and 2878 cm^−1^ coming from –CH_2_– and –CH_3_ moieties. There are also visible bands characteristic for –CO– stretching vibrations at 1006 cm^−1^. The presence of polyphenolic compounds is confirmed by the bands in the regions from 1722 cm^−1^ to 1400 cm^−1^ and around 1000 cm^−1^ typical for C=C aromatic ring stretching and –C=O stretching vibrations [29,30]. However, their intensity is low. The FTIR spectrum of the commercial extract shows bands coming from carboxyl groups at 3187 cm^−1^ of quite low intensity as well as bands at 2943 cm^−1^ and 2875 cm^−1^ that are typical for –CH_2_– and –CH_3_. A band of high intensity coming from α, β-unsaturated aliphatic ester groups is present at 1713 cm^−1^, which is typical for flavonoids. The band at 1591 cm^−1^ can be assigned to C=C aromatic ring stretching vibrations. The band at 1394 cm^−1^ comes from C=C stretching vibrations. Bands at 1183 cm^−1^ and 1089 cm^−1^ can be assigned to C–O as well as C–C stretching vibrations (phenyl carbon). The spectrum of the extract prepared using 50% ethanol solution shows very similar bands; however, these are of different intensity. The broad band at 3351 cm^−1^ is typical for hydroxyl groups. Bands corresponding to C–H, –CH_2_–, and –CH_3_ at 3009 cm^−1^, 2924 cm^−1^, and 2856 cm^−1^ suggest higher content of aliphatic chains in comparison to commercial extract. The region between 1720 cm^−1^ and 1000 cm^−1^ is typical for polyphenols. The aforementioned intense band comes from α,β-unsaturated aliphatic –C=O groups. The band at 1599 cm^−1^ comes from C=C stretching vibrations of aromatic rings. The band at 1396 cm^−1^ is typical for unsaturated double bonds. Finally, bands at 1176 cm^−1^ and 1092 cm^−1^ typical for C–O and C–C (phenyl carbon) are present. The spectrum of natural extract prepared by ethanol of higher concentration (4) shows a higher content of aliphatic chains (increased intensity of bands at 2923 cm^−1^ and 2858 cm^−1^). Importantly, the band coming from the C=C aromatic ring and C=O stretching vibrations shows a higher content of polyphenolic compounds suggesting the higher efficiency of its extraction process. The last spectrum presents extract prepared using 95% EtOH solution enhanced by ultrasounds. When comparing the spectra, it can be noticed that such an obtainment method results in the product characterized by the highest content of polyphenols which prove bands at 3308 cm^−1^ as well as at 1589 cm^−1^ [29,30].

**Figure 4:**
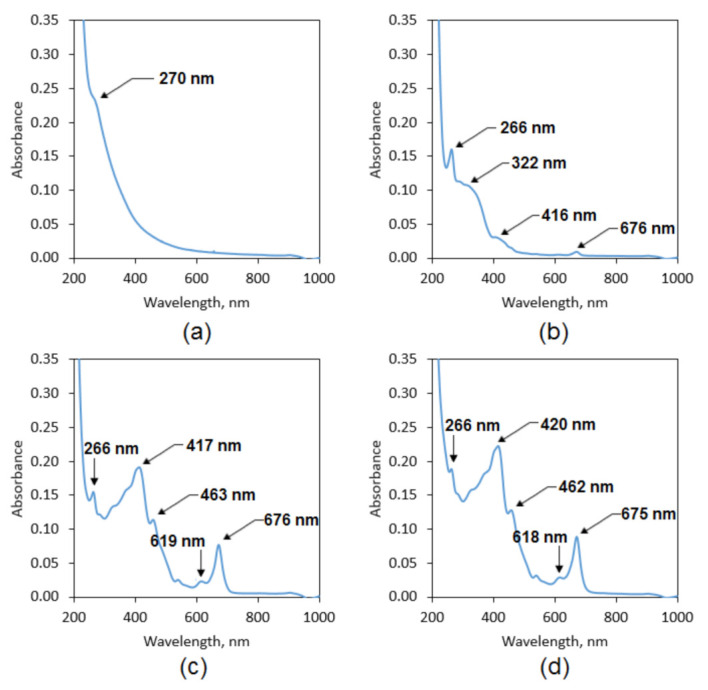
UV-Vis spectra of the natural extracts (**a**) commercially available extract; (**b**) plant extract prepared using 50% EtOH solution; (**c**) plant extract prepared using 95% EtOH solution; (**d**) plant extract prepared using 95% EtOH solution and ultrasounds.

To further investigate phenols, polyphenols, and other biologically relevant substances content, UV-Vis spectra were collected. Figure 4a shows UV-Vis spectrum of the commercial extract, which contains only one peak typical for auxochromes. The maximum peak intensity at 270 nm corresponds to nμ → π* transitions. The spectra of the extracts prepared using ethanol solutions differ from the commercial one and exhibit more peaks that are associated with the presence of the aromatic compounds, especially polyphenols. Apart from the peaks with the maximum at 266 nm (50% EtOH, 95% EtOH and 95% EtOH/ultrasounds), there are other signals typical for conjugated double bonds corresponding to *n* → π* and π → π* transitions [26].

**Figure 5:**
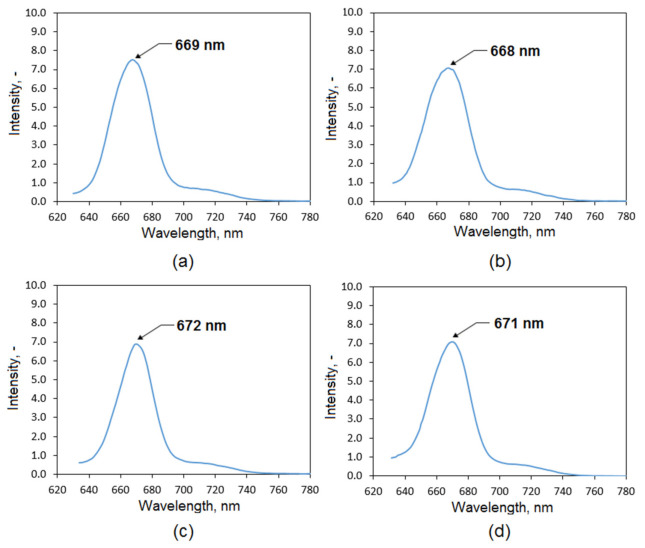
Fluorescence spectra of the natural extracts: (**a**) commercially available extract; (**b**) plant extract prepared using 50% ethanol solution; (**c**) plant extract prepared using 95% ethanol solution; (**d**) plant extract prepared using 95% ethanol solution and ultrasounds.

Figure 5 shows fluorescence spectra of the evaluated extracts. One may observe that all four samples exhibit similar peaks with the maximum at 669 nm (commercial extract), 668 nm (plant extract 50% EtOH), 672 nm (plant extract 95% EtOH), and 671 nm (plant extract 95% EtOH/ultrasounds), respectively. The signals come from porphyrin dyes present in the *Kalanchoe pinnata* Chlorophyll *a*. Their intensity is similar, which suggests comparable content.

**Figure 6:**
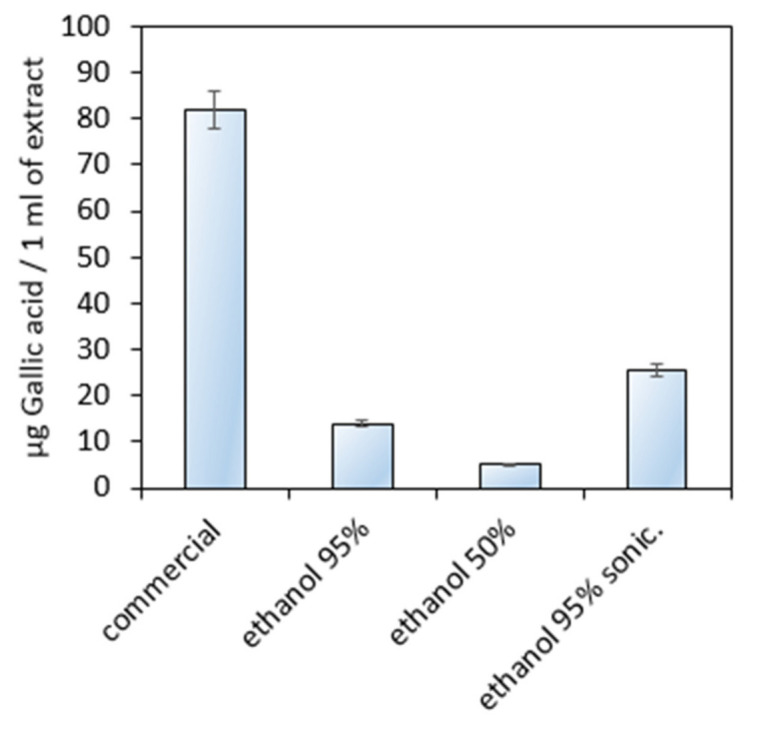
Phenols and polyphenols content investigation using Folin–Ciocalteu reagent.

To investigate the total phenolic and polyphenolic compounds content in different extracts, the UV-Vis spectroscopic method using gallic acid as a standard was applied (Figure 6), which enables quantitative determination of the aforementioned substances [27,28]. The calibration plot prepared at 750 nm resulted in R^2^ = 0.9995. The study showed that the highest quantity of the organic substances responsible for antioxidant activity of the *Kalanchoe pinnata* can be found in the commercial extract (more than 80 µg/1 mL) and can be considered as significantly different from other samples.

### 3.2. Hemostatic Agents FTIR Analysis

**Figure 7:**
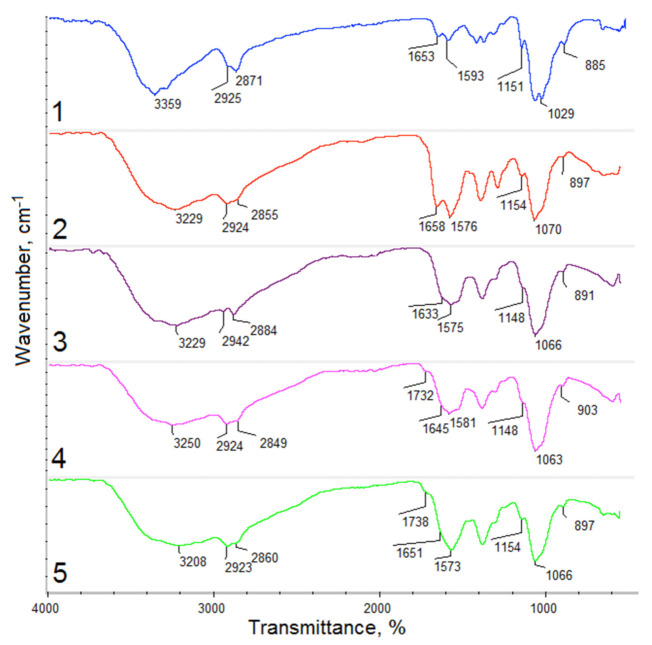
FTIR spectra of the raw fungal chitosan (**1**), Gel-1 (**2**), Gel-2 (**3**), Gel-3 (**4**), and Gel-4 (**5**) samples containing plant extract prepared using commercial *Kalanchoe pinnata* extract.

**Figure 8:**
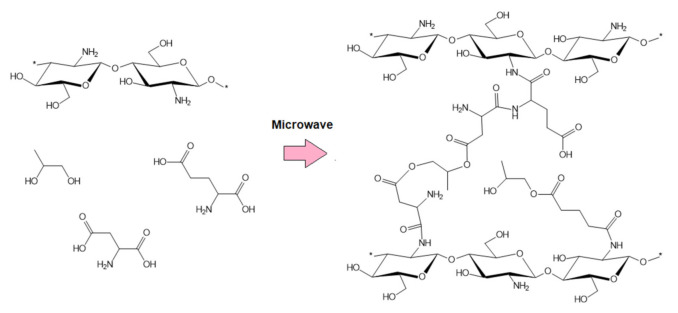
General scheme of hemostatic agent preparation (*—another polymeric unit)

FTIR spectra of the raw fungal chitosan, Gel-1, Gel-2, Gel-3, and Gel-4 samples containing plant extract prepared using commercial *Kalanchoe pinnata* extract are given in Figure 7. FTIR results are presented in Table 3. The raw chitosan spectrum shown is typical for this polymer band. First, the band coming from free hydroxyl and amino groups with the maximum at 3359 cm^−1^ is visible. The presence of NH_2_ moieties is confirmed also by the bands at 1576 cm^−1^ and 1154 cm^−1^. The band at 1653 cm^−1^ corresponds to amide bonds present in acylated units of the CS. In addition, bands coming from glycosidic bonds as well as glucopyranose rings are visible at 1029 cm^−1^ and 885 cm^−1^ [12,15,16,31]. CS crosslinked with amino acids shows some significant changes in the bands’ intensity. On the contrary to the commonly applied methods using glutaraldehyde as a crosslinking agent, there is no decrease in the free amino groups’ intensity. Since amino groups are prone to react, the crosslinking process occurs between the carboxyl groups of L-aspartic and L-glutamic acid and amino groups present in glucosamine units, due to which bands coming from free amino groups are of higher intensity compared to the native CS (1576, 1755, 1581, 1573, 1154, 1148, 1148 and 1154 cm^−1^, respectively). Importantly, when comparing collected spectra to other researchers’ data, a significant difference in amino groups content is visible [12,15,16]. Covalent bonds formation proves bands typical for amides at 1658, 1633, 1645, and 1651 cm^−1^, respectively. In each case, wide overlapping bands coming from free hydroxyl and carboxyl groups at 3229, 3229, 3250, and 3208 cm^−1^ are visible [31]. The presence of *Kalanchoe pinnata* extract confirms bands at 1732 cm^−1^ and 1738 coming from carbonyl groups as well as bands around 1400 cm^−1^ coming from unsaturated carbon double bonds as well as bands around 1600 cm^−1^, which can be assigned to C=C aromatic ring stretching vibrations [29,30]. All FTIR spectra exhibit also glycosidic bonds and glucopyranose rings typical for CS [31]. Basing on the obtained results, the proposed reaction scheme has been presented in Figure 8.

**Figure 9:**
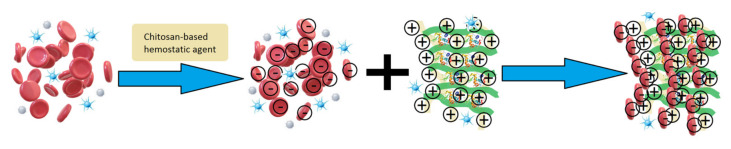
General scheme of hemostatic agent mode of action.

Raw CS is known for its favorable biological properties such as biocompatibility, biodegradability, mucoadhesiveness, antioxidant, and hemostatic activity. Importantly, most of them are related to the presence of free amino groups, which interact with negatively charged cell membranes, as given in Figure 9. Such chemical composition should provide sufficient blood components interaction and provide superior hemostatic activity comparing to other polymer-based biomaterials prepared using cellulose, poly(ethylene glycol), gelatin, and others [9].

### 3.3. Viscosity Study

**Figure 10:**
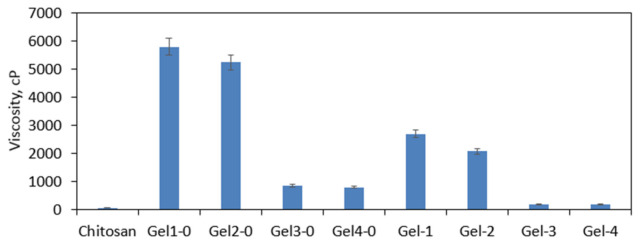
Viscosity study of the raw chitosan, hydrogels after crosslinking (Gel1-0 to Gel4-0), and hydrogels after lyophilization and swelling with distilled water (Gel-1 to Gel-4).

The chemical crosslinking resulted in the change of the CS viscosity as given in Figure 10. Native CS has a linear structure and contains *N*-acetylglucosamine as well as glucosamine mers linked with glycosidic bonds. Thus, it easily dissolves in acidic solutions whose viscosity is very low (52 cp). The crosslinking process leads to the formation of cross bindings; thus, a crosslinked structure is obtained. On the other hand, this fact makes the polymer insoluble in aqueous solutions, which instead of dissolving undergoes swelling without losing their shape and structure. To verify the effect of crosslinking conditions on the raw CS, newly prepared hydrogels directly after crosslinking were placed in the acetic acid solution, and their viscosity was determined. As one may notice, in the case of samples Gel-1-0 and Gel-2, numerous covalent bonds have been formed, leading to the highly crosslinked and tight structure formation, which resulted in the significantly higher viscosity. Samples Gel-3-0 and Gel-4-0 also were very viscous (800–900 cp). To prepare hemostatic materials, raw hydrogels were lyophilized. The products in their final form were characterized by reduced viscosity, which can be assigned in the change of their porosity and spatial architecture due to the dry-freezing process. It is noteworthy that such viscosity behavior is typical for hydrogel materials and enables distinguishing physical and chemical ones [32,33,34,35,36].

### 3.4. Swelling Properties Study

**Figure 11:**
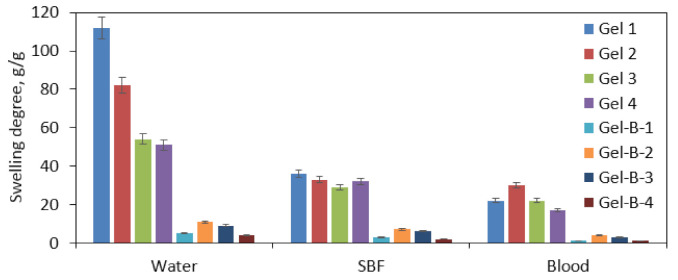
Swelling properties study in different swelling media (distilled water, simulated body fluid (SBF), and blood) of Gel 1–4 hemostatic agents in the form of a patch; Gel-B-1–4 hemostatic agents in the form of beads.

Hydrogels can be described as hydrophilic materials with a three-dimensional porous structure that can absorb high amounts of water and aqueous solutions. Therefore, their swelling abilities are correlated with the chemical structure as well as porosity and presence of interconnected channels. In this article, using the same raw materials, two different types of potential hemostatic agents have been developed, namely patches (dedicated to broad but shallow wounds) and beads (with the future applications for deep puncture and shotgun wounds). To verify their potential, a study of the swelling properties has been carried out using distilled water, SBF, and human blood (Figure 11). Samples Gel 1 and Gel 2 exhibited superior swelling capacity comparing to other samples, especially in the case of distilled water, which was considered as significantly different. However, it must be acknowledged that absorbing high amounts of the liquid may result in an undesired materials volume increase followed by its structure collapse, which may result as a consequence in the integrity disruption. Samples Gel 3 and 4 are characterized by nearly a twice lower swelling degree in distilled water, whereas this feature in SBF is very similar to the Gel 1 and Gel 2 samples. Such difference can be assigned to the crosslinking agents ratio used for hemostatic agents’ preparation. The higher the L-glutamic acid content, the higher the swelling degree that has been obtained, which can be caused by a slightly longer carbon chain providing more space for water molecules after crosslinking. There is a high difference in the sorption abilities comparing various swelling media. The swelling is much lower for SBF and blood, which can be explained by the more complexed chemical composition, especially for the last one, which contains not only various ions but also proteins and other molecules that may interact with amino or hydroxyl groups of the materials due to hydrogen bonds formation, thus hampering water molecules penetration inside the materials’ structure [31]. Additionally, there is a high dissimilarity for a patch and beads. The hemostatic agents in the form of spheres are characterized by a few times smaller swelling degrees than patches that can be explained by the lower porosity resulted due to differences in fabrication methods. Importantly, in the case of samples swollen with human blood for both forms, the best sorption abilities were obtained for sample no. 2 with the highest glutamic acid content. The sorption properties can be assigned to the presence of multiple hydrophilic moieties present in the modified CS structure as well as incorporated additional groups such as amino and carboxyl ones [31,37]. It is noteworthy that incubation for all samples lasted 5 min, which leads to the conclusion that all samples in such a short period of time are capable of stopping the bleeding and forming an artificial clot [9].

### 3.5. Antioxidant Activity Study

**Figure 12:**
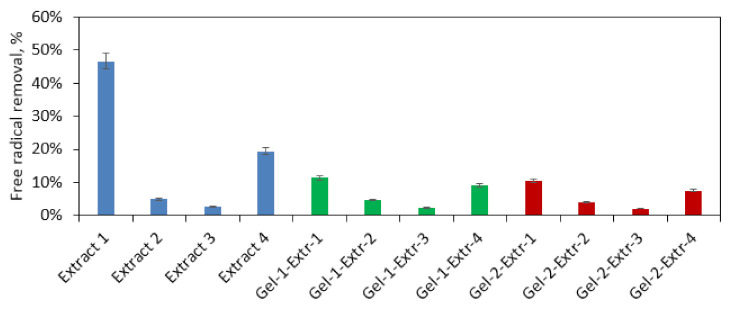
Antioxidant properties study by (2,2-diphenyl-1-picrylhydrazyl) (DPPH) method.

The formation of a wound as a result of skin damage may lead to oxidation stress, which negatively affects the healing process. Therefore, additional biological activity leading to the free radical scavenging is a highly desired feature for modern biomaterials. HemA modified with four different extracts was investigated for its antioxidant activity (Figure 12). The performance of Extracts 1 and 4 as well as samples Gel-1-Extr-4 and Gel-3-Extr-1 can be considered as significantly different. The results given in Figure 3, Figure 4 and Figure 5 have shown that all of them contain phenolic and polyphenolic compounds responsible for free radicals’ neutralization. To verify this feature, the extracts and two samples characterized by best swelling capacity (Gel 1 and Gel 2) were used to study their antioxidant behavior. The highest biological activity was obtained for commercial extract followed by the extract prepared using 95% ethanol solution subjected to ultrasounds treatment, which were able to remove almost 50% of DPPH• (Extract 1) and nearly 20% (Extract 4), respectively in only one hour. Further tests were carried out for extracts immobilized inside hemostatic agents’ matrixes. One may observe that after 60 min, biomaterials have neutralized more than 10% of free radicals (Extract 1 and 4). For Extracts 2 and 3, the amounts of free radicals being removed were similar for the solutions being tested alone and for ready biomaterials. *Kalanchoe pinnata* is a plant known for its antioxidant activity, which can be assigned to the phenolic and polyphenolic compounds present in its leaves [24]. On the other hand, CS due to the free amino groups as well as secondary hydroxyl moieties content also possesses the ability of free radicals’ removal. The significantly higher antioxidant performance of the raw extract is explained by the easier access to DPPH•, which is much hampered due to the strict and compact form of the hemostatic agents. However, comparing evaluated biomaterials activity to similar products, the antioxidant activity is considerably higher [31].

### 3.6. Morphology Study

**Figure 13:**
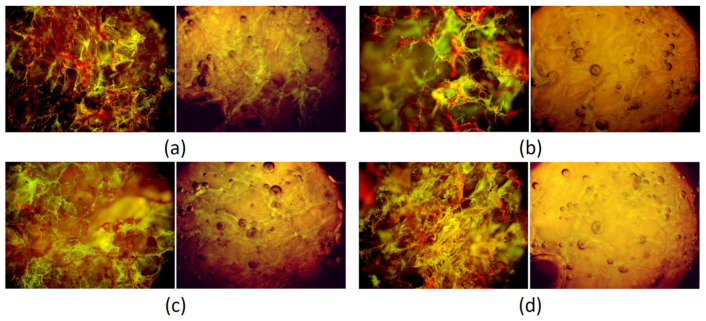
Microphotographs of the hydrogels (**a**) Gel-1 dried and swollen; (**b**) Gel-2 dried and swollen; (**c**) Gel-3 dried and swollen; (**d**) Gel-4 dried and swollen.

Prepared gels in the patches form were investigated for their morphology in a dried and swollen form (Figure 13). One may notice that all samples are highly porous and possess numerous interconnected channels that easily fill with the swelling medium. It is noteworthy that due to their superior porosity and a lot of free space inside the 3D matrix after contact with the hydrophilic liquid, they do not increase their volume in a significant manner, since it replaces the air present inside the channels.

**Figure 14:**
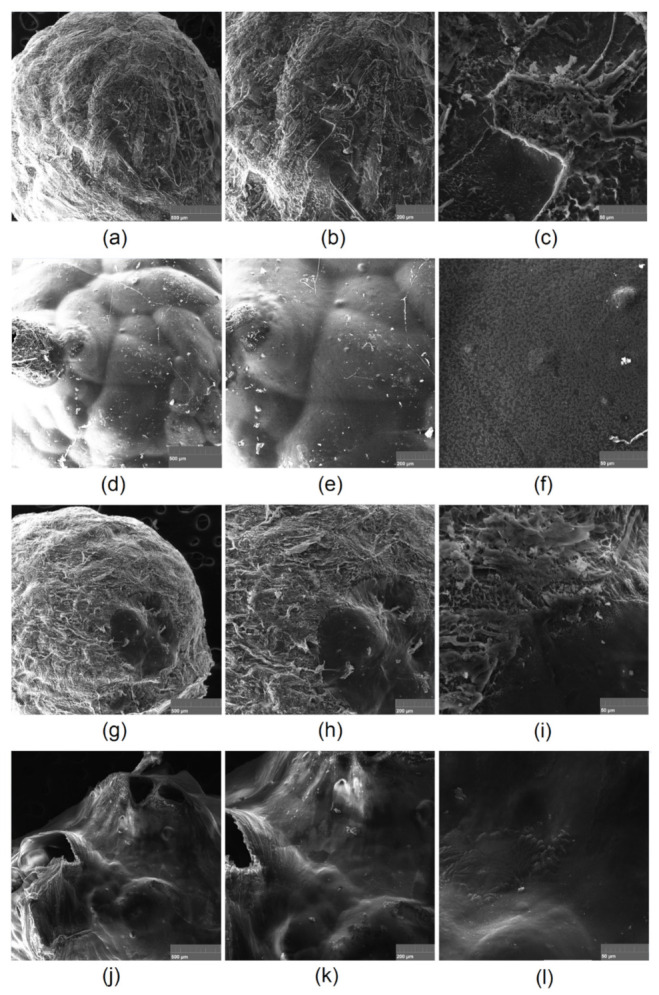
SEM microphotographs of the chitosan-based beads: (**a**) Gel-1-B 500×; (**b**) Gel-1-B 1000×; (**c**) Gel-1-B 5000×; (**d**) Gel-2-B 500×; (**e**) Gel-2-B 1000×; (**f**) Gel-2-B 5000×; (**g**) Gel-3-B 500×; (**h**) Gel-3-B 1000×; (**i**) Gel-3-B 5000×; (**j**) Gel-4-B 500×; (**k**) Gel-4-B 1000×; (**l**) Gel-4-B 5000×.

Figure 14 presents SEM microphotographs of the hemostatic agents in the form of beads. All spheres are characterized by a dense surface. Samples Gel-1-B and Gel-3-B under higher magnification exhibit some roughness, whereas Gel-2-B and Gel-4-B surfaces are smoother but contain “volcano-like” regions, which can be a consequence of the air leaving the bead after a coagulation bath. The lower amount of macropores in a dried form compared to the patch materials explains the lower swelling abilities.

**Figure 15:**
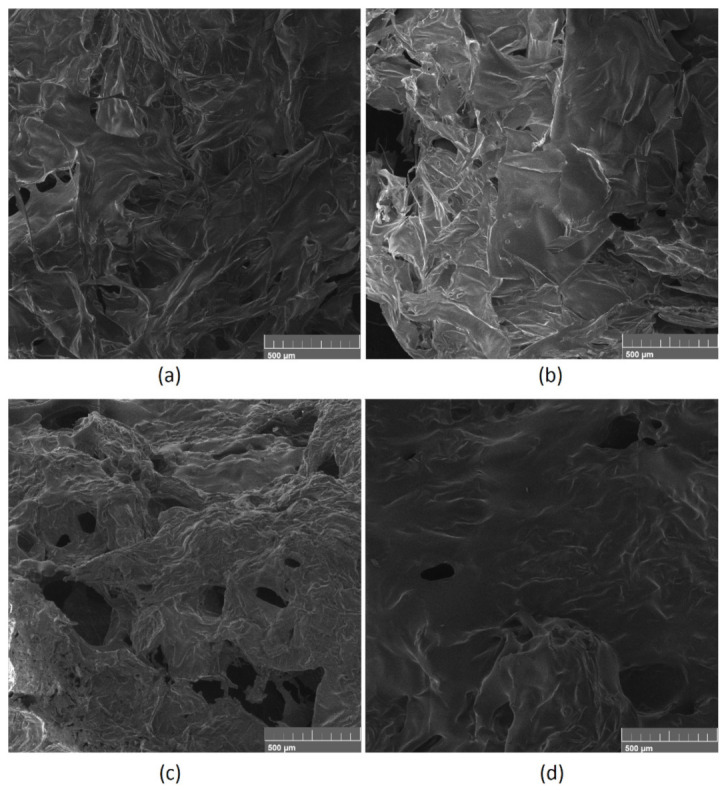
SEM microphotographs showing surface roughness of (**a**) Gel-1, (**b**) Gel-2, and (**c**) Gel-1 after incubation with blood; (**d**) Gel-2 after incubation with blood.

The samples with highest ability of blood sorption were compared before (Figure 15a,b) and after contact with the swelling medium (Figure 15c,d). Both samples Gel-1 and Gel-2 were characterized by a spatial, highly porous structure with high curvature and multiple interconnected channels, which highly contribute to excellent swelling properties, as shown in Figure 11. The pores edges are quite sharp and of petal-like shape. The presence of macropores enables aqueous blood fraction migration inside the three-dimensional structure as well as its stabilization inside the biomaterial. There are no significant differences between Gel-1 and Gel-2, which leads to the conclusion that the chemical composition plays a major role in the swelling capability. Figure 15c,d shows the materials surface after incubation with human blood. It is noteworthy that some significant changes may be observed. The surfaces are smoother and covered with protein-like substances. It can be noticed that the surface coverage is higher in the case of Sample Gel-2, which leads to the conclusion that this material interacts stronger with blood components due to electrostatic attractions between positively charged amino groups and negatively charged carboxylic ones. Almost all pores are covered with biomolecules. Such results show that the hemostatic agents 5 min after placement may achieve nearly full sorption capacity, thus potentially blocking the hemorrhage, which is a very important feature for the future applications.

**Figure 16:**
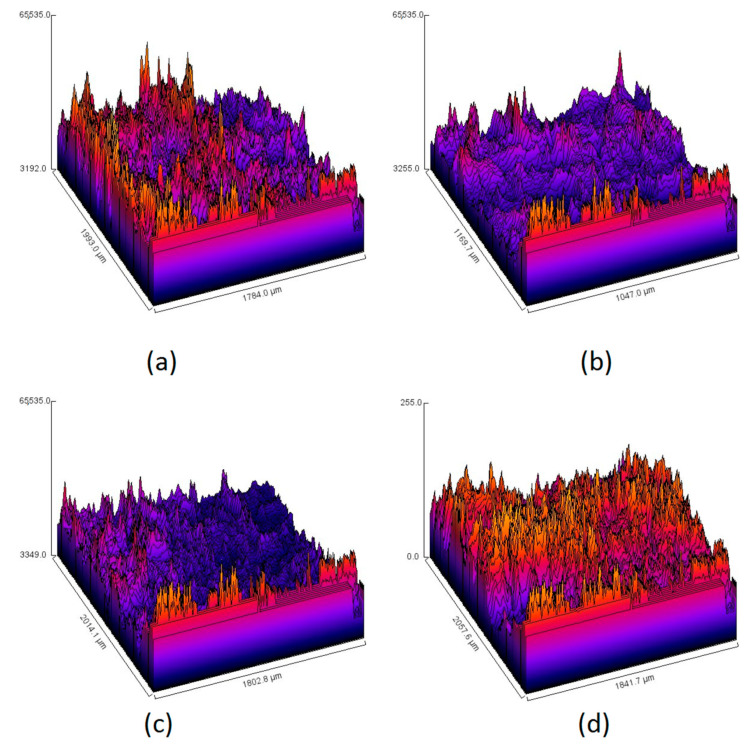
Surface roughness of the Gel-1 and Gel-2 samples before (**a**,**b**) and after incubation with blood (**c**,**d**).

Figure 16 shows the samples roughness before (a, b) and after (c, d) incubation with blood. Both samples before the incubation are characterized by very high roughness due to the numerous open-structure pores and their sharp edges. After contact with human fluid due to the material coverage with blood components, the surface is significantly smoother, and the roughness has decreased. It can be observed that the interactions between the swelling medium and material are a little bit different, since it seems that the adhesion is higher in the case of the Gel-2 sample. The sample Gel-1 surface is much smoother after blood contact. The change in the materials surface can be explained by the electrostatic effects leading to the negatively charged molecules attraction and immobilization resulting in the artificial clot formation. Such behaviors are explained by the chemical structure of the CS, which contains free amino groups that interact with erythrocytes and platelets [9,10,31]. The incorporation of L-aspartic (C5) and L-glutamic acid resulted in the increase of NH_2_ moieties which, on the other hand, could lead to the increased electrostatic attractions between negatively charged blood components such as proteins and cells, which is crucial during blood clotting cascade and hemostasis restoration [9].

### 3.7. Mechanical Properties Study

**Figure 17:**
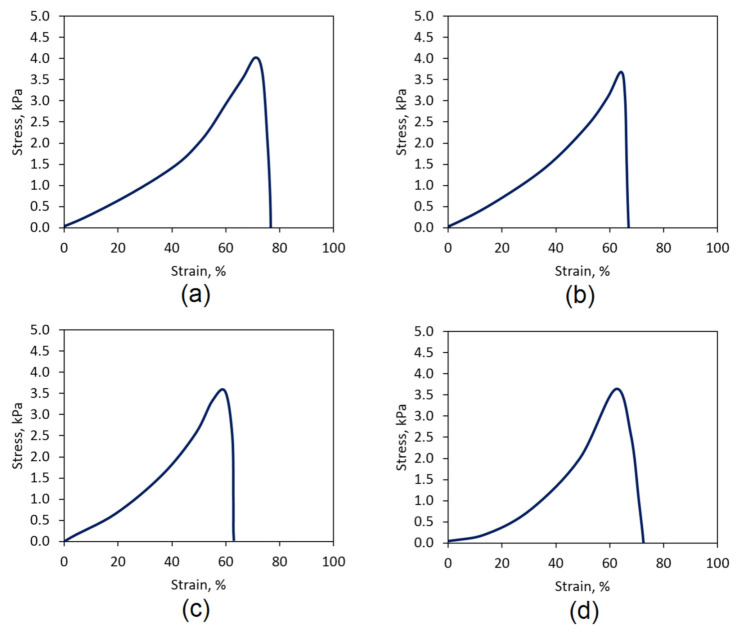
Mechanical durability of the samples: (**a**) Gel-1; (**b**) Gel-2; (**c**) Gel-3; (**d**) Gel-4.

Biomaterials dedicated for topical applications should be characterized with appropriate mechanical properties to maintain their integrity after swelling with body fluids and not tearing to release smaller fragments, which may contaminate the wound. Hydrogels depending on the polymer used for their preparation are characterized by varied durability. Natural raw materials are mostly characterized by lower resistance to mechanical factors than synthetic ones. Figure 17 shows the results of TS evaluation performed on the “dog bone”-shaped samples swollen with SBF. Raw CS is characterized by low endurance. The crosslinking process leading to the formation of bridges from *L*-aspartic and *L*-glutamic acid molecules between linear chains leading to crosslinked structure formations enhances this feature. A crucial factor affecting the increased durability is the formation of covalent amide bonds between free amino groups coming from CS and carboxylic moieties present in amino acids. Figure 17 shows the tensile strength for the Gel-1, Gel-2, Gel-3, and Gel-4 samples. The highest mechanical durability was obtained for the sample Gel-1, whose TS was above 4 kPa and had a strain of almost 80%. Other samples’ TS value were slightly below 4 kPa. Such results correspond to other researchers’ data [38,39,40,41] and suggest that the prepared biomaterials durability is at the satisfactory level for topical hemostatic agents’ applications.

### 3.8. Cytotoxicity Study

**Figure 18:**
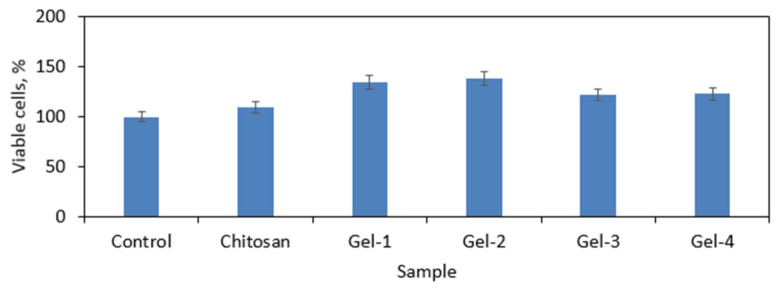
Cytotoxicity study performed using (2,3-Bis-(2-Methoxy-4-Nitro-5-Sulfophenyl)-2H-Tetrazolium-5-Carboxanilide) (XTT) assay.

To investigate in vitro biocompatibility of the prepared materials by the quantitative method, XTT assay was carried out, which is a standard procedure for medical devices. According to the ISO 10993 norm, the biomaterial is non-cytotoxic when the number of viable cells is not lower than 70% compared to the control. CS is well-known for its biocompatibility, which can be assigned to the structural similarities to glycosaminoglycans such as chondroitin sulfate, which is one of the natural ECM components. It can be observed that the presence of pristine CS has a positive impact on cells’ proliferation (Figure 18). It is noteworthy that the chemical modification of this biopolymer structure may deteriorate this property, which can be e.g., a result of toxic crosslinking agents’ release to the cell culture medium. Figure 18 shows that all of the prepared samples do not cause any negative effect on the number of viable cells. On the contrary, in the case of samples Gel-1 and Gel-2, the number of alive cells is higher by almost 50%. The viable percentage of the Gel-3 and Gel-4 samples was slightly lower yet still higher than that of native CS [31,42].

**Figure 19:**
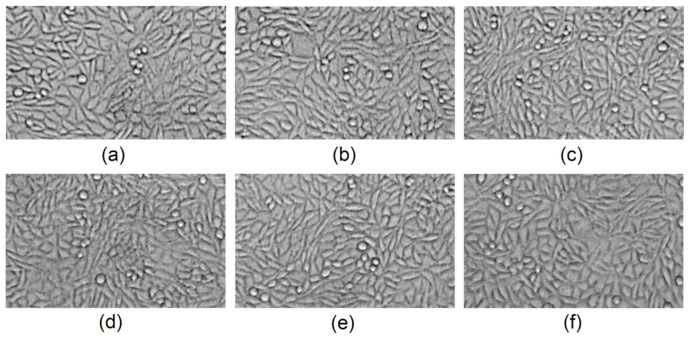
Fibroblasts’ morphology study: (**a**) control; (**b**) raw chitosan; (**c**) Gel-1; (**d**) Gel-2; (**e**) Gel-3; (**f**) Gel-4.

The lack of cytotoxicity was further studied by cells’ morphology investigations (qualitative method) after 72 h of cell culture. As shown in Figure 19, after 3 days, the cells formed monolayers. The fibroblasts were flattened and adhered to the flask bottom. The cells were characterized by a spindle-like shape typical for L929. There are a few rounded cells present. No detached fibroblasts are visible. Therefore, the biomaterials can be described as non-cytotoxic to the skin cells [31,42].

### 3.9. Future Perspectives

Performed studies showed that the application of fungal CS and other natural components led to the preparation of novel biomaterials with interesting properties. The choice of non-animal raw materials increases their potential use to patients suffering from allergy to shellfish. Importantly, the products can be obtained from cheap and locally available resources. Due to the satisfying biological features of the potential hemostatic agents, further actions will be focused on in vivo tests on small animal models. The positive effect on skin cells suggests that future application should mostly manage superficial hemorrhages, since the biomaterial may contribute to faster healing. On the other hand, obtainment of the products in the spherical shape gives the possibility of their use in deep gunshot or stab wounds. Before their commercial application, the biomaterials should undergo upscaling, which can be a challenging process due to the risk of local overheating spots appearing during the crosslinking procedure, which is a common problem associated with microwave-assisted synthesis. This phenomenon may lead to some problems with the repeatability of the samples and their homogeneity. Another issue related to microwaves is their penetration depth, which must be taken under consideration when choosing an appropriate reactor type. One possible limitation is also the risk of insufficient accessibility of the raw materials, which would meet all of the requirements for medical devices, since currently commercially available CS-based biomaterials are produced mostly using shellfish as a substrate, not fungi. However, the choice of an appropriate reaction vessel as well as stirring method probably will help to overcome this issue.

## 4. Conclusions

The aim of the following research was to obtain novel naturally derived blood clotting agents in the form of dressing and beads using fungal chitosan as a starting material, which was subjected to successful crosslinking with amino acids under microwave-assisted conditions followed by modification with four different *Kalanchoe pinnata* extracts to increase the antioxidant activity of the potential hemostatic biomaterials. Their shape was successfully processed to both patch and beads forms. Moreover, the products retained favorable features of native chitosan. The resulted products were investigated for their chemical structure and composition, confirming increased free amino groups content and phenolic/polyphenolic compounds presence. A morphology study showed spatial and porous architecture responsible for sorption capacity. The biomaterials in form of the patch exhibited superior swelling properties after incubation with human blood, whose components have been proven to interact with the evaluated samples surface. They exhibited satisfactory mechanical durability. Their ability of free radicals scavenging was proved by the DPPH method. Their lack of cytotoxicity confirmed by XTT assay and morphology study shows their potential in the future applications as hemostatic agents.

## Figures and Tables

**Table 1:** Samples description and composition.

Sample	Glu:Asp, g:g	Lyophilization	Form
Gel1-0	0.90:0.10	No	Patch
Gel2-0	0.75:0.25	No	Patch
Gel3-0	0.25:0.75	No	Patch
Gel4-0	0.10:0.90	No	Patch
Gel-1	0.9:0.10	Yes	Patch
Gel-2	0.75:0.25	Yes	Patch
Gel-3	0.25:0.75	Yes	Patch
Gel-4	0.10:0.90	Yes	Patch
Gel-1-B	0.9:0.10	Yes	Beads
Gel-2-B	0.75:0.25	Yes	Beads
Gel-3-B	0.25:0.75	Yes	Beads
Gel-4-B	0.10:0.90	Yes	Beads

**Table 2:** Fourier transform infrared spectroscopy (FTIR) analysis results.

Sample	O–H	C–H	C=C–H	C=O	C=C	C–O
	cm^−1^	cm^−1^	cm^−1^	cm^−1^	cm^−1^	cm^−1^
*Kalanchoe pinnata* leaf	3324	2923	-	1722	1429	1006
		2878				
Commercially available extract	3187	2943	-	1713	1591	1118
		2875			1394	
					1089	
Plant extract prepared using 50% ethanol solution	3351	2924	3009	1720	1599	1176
		2856			1396	
					1092	
Plant extract prepared using 95% ethanol solution	3304	2923	3009	1721	1601	1173
		2858			1386	
					1074	
Plant extract prepared using 95% ethanol solution and ultrasounds	3308	2975	-	1714	1589	1236
		2925			1394	
					1076	

**Table 3:** FTIR results.

Sample	O–H	C–H	C=O	–NH_2_	C–O	–COO–
	cm^−1^	cm^−1^	cm^−1^	cm^−1^	cm^−1^	cm^−1^
Raw fungal chitosan	3359	2925	1653	1593	1029	-
		2871		1151	885	
Gel-1	3229	2924	1658	1576	1070	-
		2855		1154	897	
Gel-2	3229	2924	1658	1575	1066	-
		2284		1148	891	
Gel-3	3250	2924	1645	1581	1063	1732
		2849		1148	903	
Gel-4	3208	2923	1651	1573	1066	1738
		2860		1154	897	

## Data Availability

The data presented in this study are available on request from the corresponding author.
